# Supplementary material for: The effect of self-organizing map architecture based on the value migration network centrality measures on stock return. Evidence from the US market
Source: PLoS One. 2022 Nov 1;17(11):e0276567. doi: 10.1371/journal.pone.0276567 (PMC9624434; doi:10.1371/journal.pone.0276567)
Supplement: S1 File — Evidence from the US market. The Supporting Information, S1 File, contains additional tables and graphs for the robustness analysis. (PDF) [file pone.0276567.s001.pdf]

Supporting information for: The effect of self-organizing map architecture based on the value migration network centrality measures on stock return. Evidence from the US market.

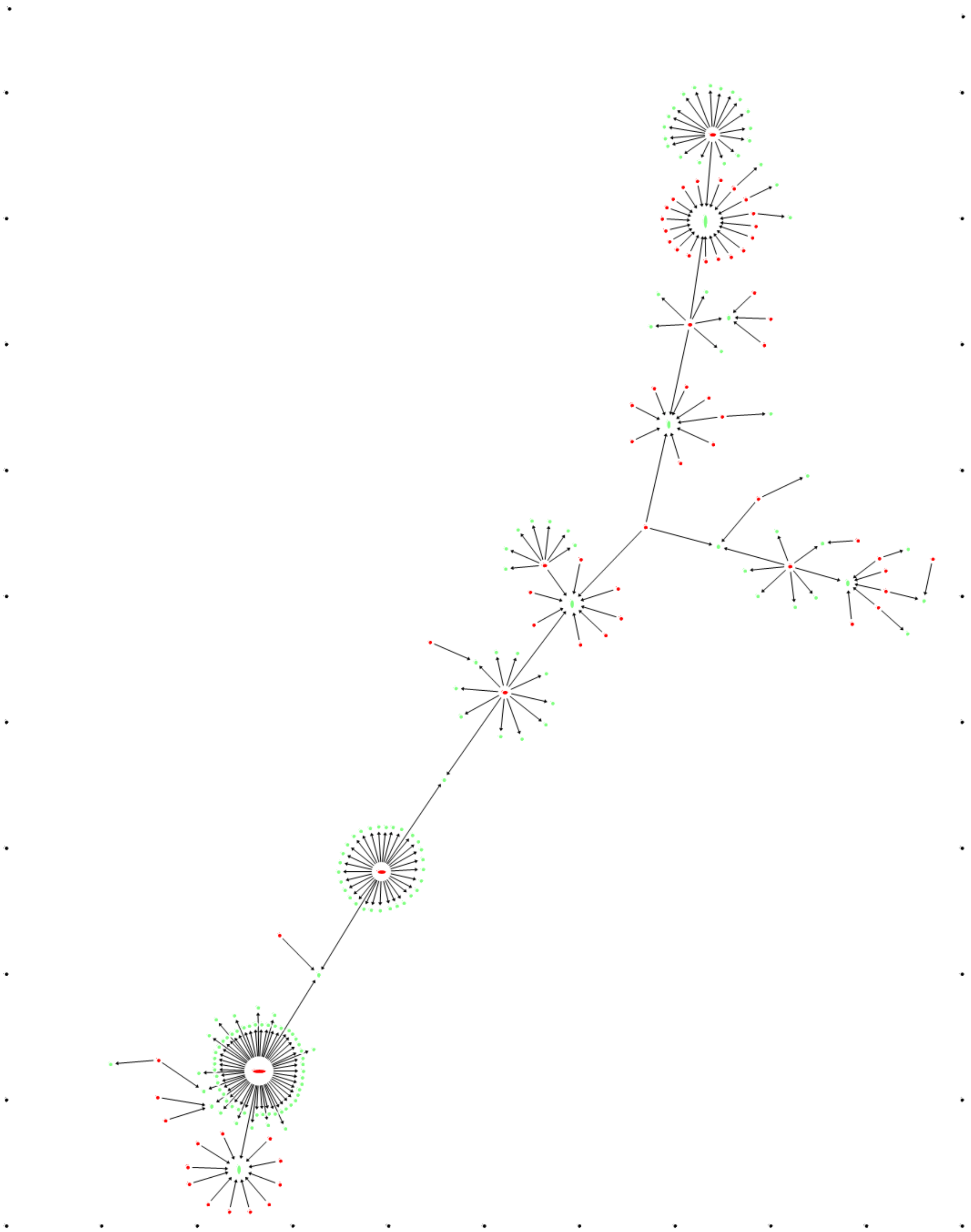

Fig A1. Value migration network for the 250 largest companies from the S&P500 index. Green nodes denote companies in the inflow stage; red vertices indicate firms in the outflow stage; black nodes are isolated (visualization algorithm: Fruchterman & Reingold)

Table A1. Representation of the 219 companies from the largest component of the VMN in SOM 3 x 4 for specific datasets

Sample:	Training, Test, Validation			
Cluster position	1	2	3	4
1	3	14	0	56
2	52	0	0	3
3	0	80	7	4

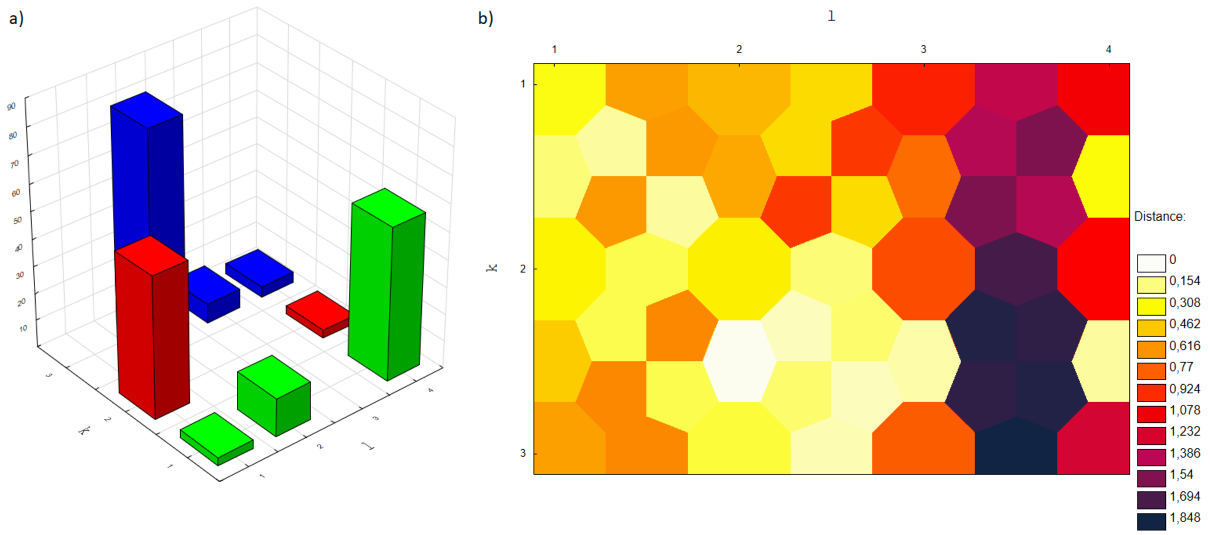

Fig A2. The numbers of firms classified into disjoint clusters (a) and the distances between neurons (b) for SOM 3 x 4 (219 companies from the largest component of the network for the 250 largest companies)

Table A2. Average value of ARRS for individual neurons and their ordering (219 companies from the largest component of the network for the 250 largest companies)

Neuron	n	Proportion	Cumulative Proportion	Mean $ARRS_i$	Cluster of centrality (CC_i)
**(1, 1)**	3	0.014	0.014	-0.0401	1
**(1, 2)**	14	0.064	0.078	0.0367	2
**(2, 1)**	52	0.237	0.315	0.0952	3
**(3, 3)**	7	0.032	0.347	0.2694	4
**(3, 2)**	80	0.365	0.712	0.3059	5
**(1, 4)**	56	0.256	0.968	0.3371	6
**(2, 4)**	3	0.014	0.982	0.3372	7
**(3, 4)**	4	0.018	1.000	0.3385	8

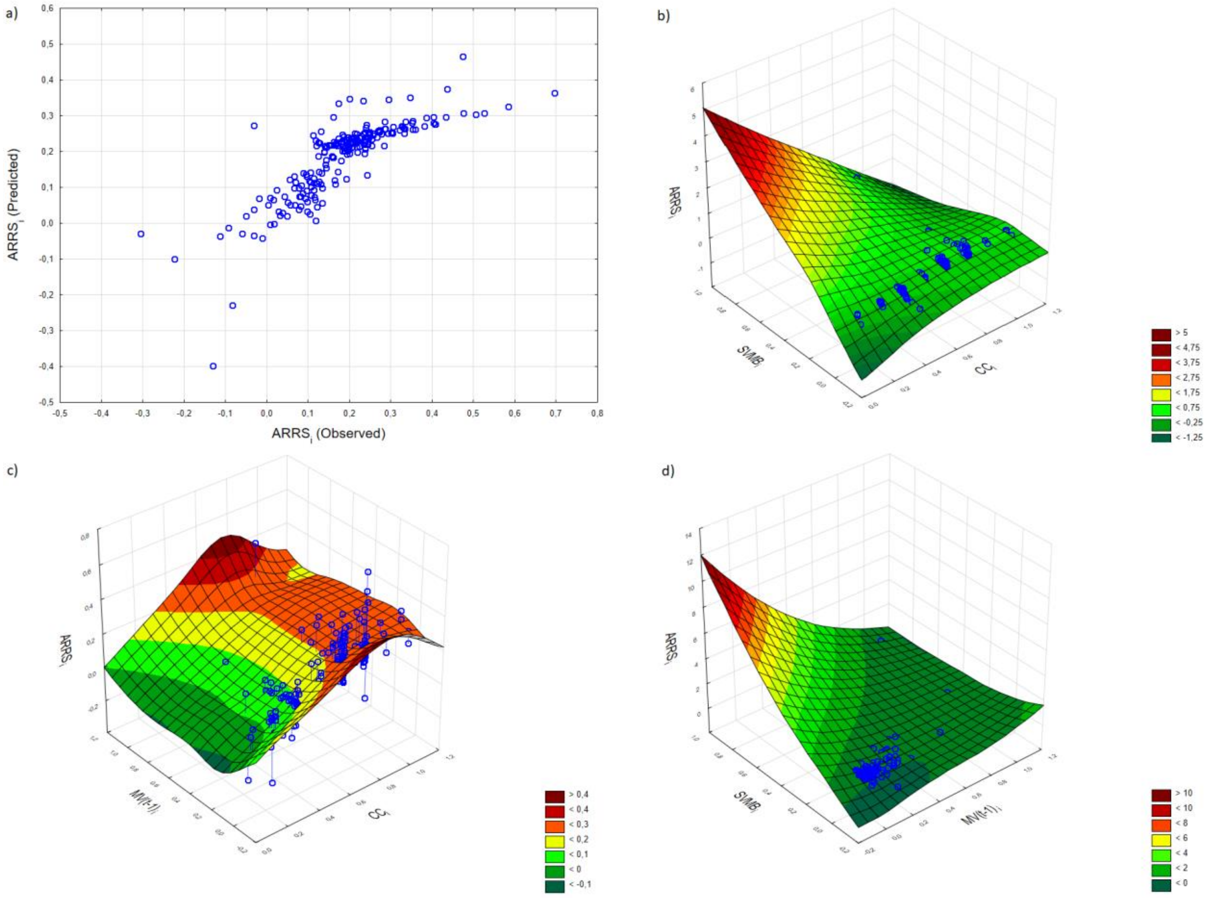

Fig A3. Graphical illustration of MARS model 5. (a) ARRS values observed versus predicted; (b) first-order term of the predictor variables CC_i and $SVMB_i$; (c) first-order term of the predictor variables CC_i and $MV_{(t-1)}$; (d) first-order term of the predictor variables $MV_{(t-1)}$ and $SVMB_i$

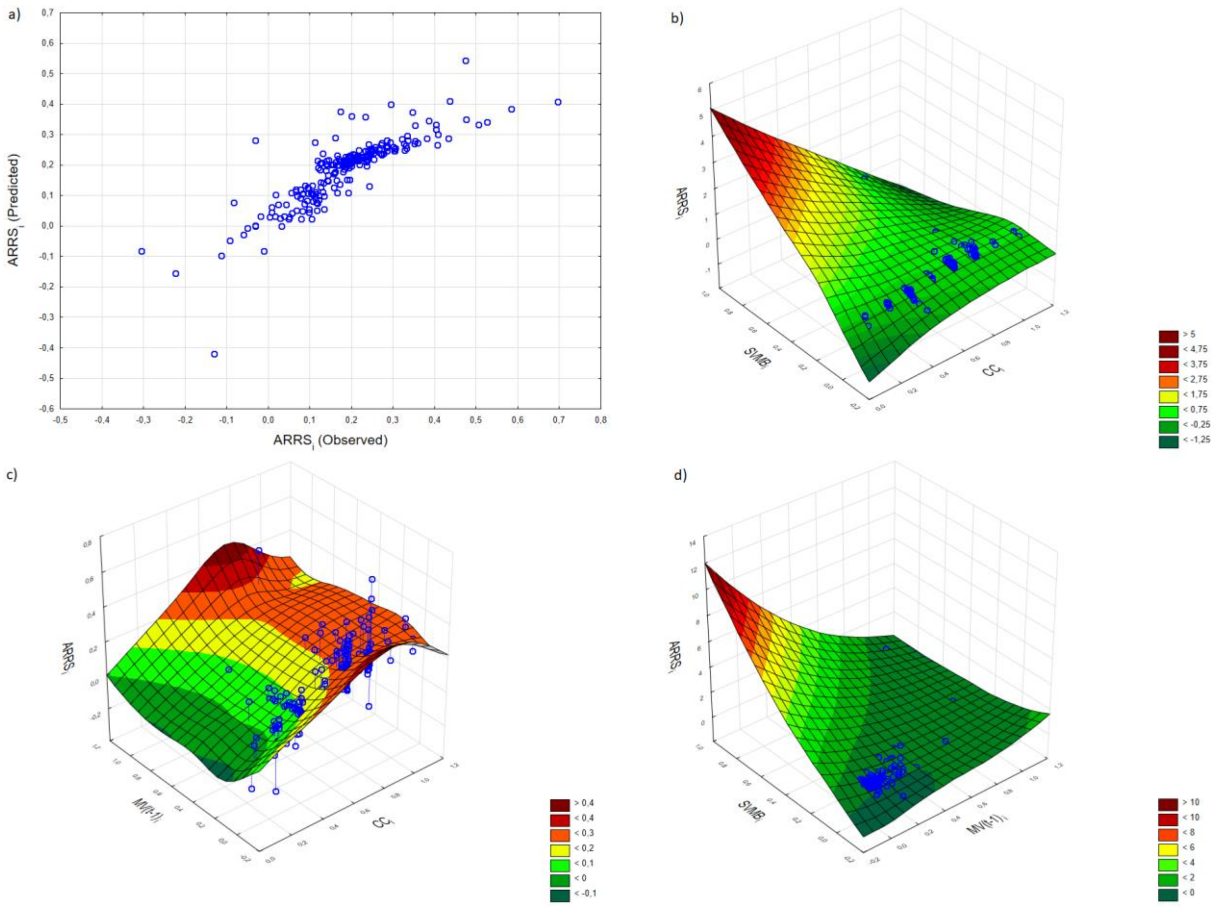

Fig A4. Graphical illustration of MARS model 6. (a) Observed versus predicted ARRS values; (b) second-order term of the predictor variables CC_i and $SVMB_i$; (c) second-order term of the predictor variables CC_i and $MV_{(t-1)}$; (d) second-order term of the predictor variables $MV_{(t-1)}$ and $SVMB_i$
