# Supplementary material for: The effect of self-organizing map architecture based on the value migration network centrality measures on stock return. Evidence from the US market
Source: PLoS One. 2022 Nov 1;17(11):e0276567. doi: 10.1371/journal.pone.0276567 (PMC9624434; doi:10.1371/journal.pone.0276567)
Supplement: Attachment — Submitted filename: Detailed Response to Reviewers.docx [file pone.0276567.s003.docx]

**Detailed Response to Reviewers**

**Response to Academic Editor**

September 26, 2022

*Dear Academic Editor,*

Thank you very much for the referee report on my manuscript PONE-D-22-15643 entitled “The effect of self-organizing map architecture based on the value migration network centrality measures on stock return. Evidence from the US market”. I have revised the paper to fully address all Reviewer comments.

Please find enclosed a detailed, point-to-point response to all Reviewer comments with all changes clearly specified. Changes in the text are marked with colors, as follows: Reviewer #1: green; Reviewer #2: yellow. Minor inaccuracies have been found during editing and correction. They have been rectified and marked in blue. All page references are related to the manuscript with the marked changes.

I wish to take this opportunity to thank the Reviewers for their time, effort and insightful comments that have resulted in an improved manuscript. I would also like to thank you for handling my paper. Hopefully, my revised manuscript can be regarded as meeting the high standards of PLOS ONE journal.

With kind regards

**Point-to-point responses to the Reviewers**

**Response to Reviewer #1:**

I am grateful for the comments provided by the Reviewer to improve my manuscript. In my new version, I have followed the recommendations and your comments have been fully addressed. Text changes are marked in green. The newly added and significantly revised portions in the paper are also marked in green to make them easy to identify. Minor inaccuracies have been found during editing and correction. They have been rectified and marked in blue. All page references are related to the manuscript with the marked changes.

**Comment 1:** “*In this manuscript the network measures are used for studying the value migration process based on the stock data of 498 companies listed in the S&P500 index between the end of 2018 to the end of 2019 which results in 253 daily closing prices in each time-series. The first related question is why just this period? Even more importantly, however, using such a collection of 498 time series of length 253 results in a 498 X 498 correlation matrix (introduced in section 2.1) whose real rank is 253 thus produces 498-253 = 245 zero modes. Expressing this in simple terms such a collection of data points is too poor to project out the full complexity of correlations among the 498 companies. This makes the results presented unreliable.*”

**Reply:** The value migration process should be analyzed over a properly defined period. It should not be too short in order to eliminate short-term fluctuations in stock prices. However, it should not be too long either to capture the scale of the process of changing the value of companies. The adopted period of one year results from the need to capture the current process of value migration. Given the importance of the Reviewer’s remark, additional analyzes were carried out for the same period, using the same methodology and research procedure, but with a limited research sample of the 250 largest companies from S&P500. Additional tests carried out demonstrated that the main findings are robust to the revised research sample. A similar approach was used in the study (Coronnello, C., Tumminello, M., Lillo, F., Miccichè, S., & Mantegna, R. N. (2007). Economic sector identification in a set of stocks traded at the New York Stock Exchange: a comparative analysis. Noise and Stochastics in Complex Systems and Finance, 6601, 66010T. <https://doi.org/10.1117/12.729619>) to investigate the statistical properties of price returns for the 100 largest companies on the NYSE in one year, 2002.

**Pages 35-38: A new Section “5. Additional analysis” has been added.**

***5. Additional analysis***

*In this section, the robustness of obtained results is carried out to narrow the research sample to the 250 largest companies from the S&P500 index. The largest companies in terms of market capitalization in 2019. The analysis period remains the same; therefore, the dataset consists of 253 daily closing prices in every 250 time series. The value migration network for the 250 largest companies is presented in Figure A1, available in Supporting Information, S1 File. The largest component of the VMN contains 219 firms.*

*The obtained topological layer of the SOM with the assumed dimensions of 3 x 4 again contains eight disjoint groups. The number of companies assigned to individual clusters is presented in Table A1 and Fig A2a (see S1 File. Supporting information). The calculation of the cluster of centrality (CC_i_) variable is provided in Table A2 (see S1 File. Supporting information).*

*The results of the MARS regression without the interaction of variables (model 5) are presented in Table 8.*

***Table 8. Parameters and results for model 5***

| MODEL 5 | | | | |
| --- | --- | --- | --- | --- |
| Parameters | | **Value** | | |
| Dependent variable | | *ARRS_i_* | | |
| Independent variables | | *CC_i_; SVMB_i_; MV_(t-1)_* | | |
| Maximum number of basis functions | | 14 | | |
| Order of interaction | | 1 | | |
| Penalty | | 2 | | |
| Threshold | | 0.0005 | | |
| Removal of irrelevant basis functions | | Yes | | |
| Number of observations | | 219 | | |
| Basis function | | ***β*** | ***t* statistics** | ***p*** |
|  | Constant | 0.221 (0.013) | 16.854*** | 0.0000 |
| BF1 | max(0, *CC_i_* – 0.714) | 0.085 (0.063) | 1.343* | 0.0487 |
| BF2 | max(0, 0.714 – *CC_i_*) | -0.264 (0.042) | -5.834*** | 0.0000 |
| BF3 | max(0, *SVMB_i_* – 0.033) | 1.747 (0.666) | 2.622** | 0.0094 |
| BF4 | max(0, 0.033 – *SVMB_i_*) | -3.504 (0.408) | -8.592*** | 0.0000 |
| BF5 | max(0, *MV_(t-1)_* – 0.076) | -0.227 (0.061) | -3.696*** | 0.0003 |
| BF6 | max(0, 0.076 – *MV_(t-1)_*) | 1.925 (0.298) | 6.466*** | 0.0000 |
| BF7 | max(0, *SVMB_i_* – 0.064) | -1.267 (0.675) | -1.877* | 0.0491 |
| Results | | **Value** | | |
| GCV | | 0.0061 | | |
| *R^2^* | | 0.6755 | | |
| Adjusted *R^2^* | | 0.6631 | | |

standard error in parentheses; ***, ** and * denote significant levels at 0.1%, 1%, and 5%, respectively

*The final regression equation of model 5 is as follows:*

*ARRS_i_ = 0.221 + 0.085 ∙ max(0, CC_i_ – 0.714) – 0.264 ∙ max(0, 0.714 – CC_i_) + 1.747 ∙*

(43)

*max(0, SVMB_i_ – 0.033) – 3.504 ∙ max(0, 0.033 – SVMB_i_) – 0.227 ∙ max(0, MV_(t-1)_ – 0.076) + 1.925 ∙ max(0, 0.076 – MV_(t-1)_) – 1.267 ∙ max(0, SVMB_i_ – 0.064)*

*The graphical presentation of model 5 is demonstrated in Fig A3 (S1 File. Supporting information).*

*Model 5 is the counterpart of Model 1. By comparing both models, it can be seen that the structure of all the spline basis functions and the signs (positive/negative) of the regression coefficients are preserved. Furthermore, all regression coefficients of the contained spline basis functions are statistically significant (p<0.05). The value of the knot point of BF1 and BF2 is the same as in the original model: the value of 0.714 normalized knot; the value of 5 CC_i_ variable. This means that the critical threshold of the cluster for the non-linear effect remains unchanged. However, due to the change in the research sample, the knot point values have changed for the remaining functions related to the SVMB_i_ and MV_(t-1)_ variables.*

*The results of Model 6 with second-order interactions between predictors are shown in Table 9.*

*Model 6 is the counterpart of Model 2. It consists of fewer base functions, six functions related to three variables, and one function with a second-order interaction. The following basic functions of model 6 (BF1÷BF7) are equivalent to the basic functions of BF1, BF2, BF3, BF4, BF9, BF10, and BF8 of model 2, respectively. The construction of all basis functions and the signs of the regression coefficients are preserved and statistically significant at the significance level of 5%. In this case, the value of the knot point of BF1 and BF2 is the same as in the original model 2* *and for models 1 and 5.*

*The final regression equation of model 5 is as follows:*

*ARRS_i_ = 0.240 + 0.085 ∙ max(0, CC_i_ – 0.714) – 0.169 ∙ max(0, 0.714 – CC_i_) + 0.710 ∙ max(0, SVMB_i_ – 0.033) – 6.612 ∙ max(0, 0.033 – SVMB_i_) – 0.331 ∙ max(0, MV_(t-1)_ – 0.076) + 2.776 ∙ max(0, 0.076 – MV_(t-1)_) + 19.963 ∙ max(0, MV_(t-1)_ – 0.027) ∙*

(44)

*max(0, 0.033 – SVMB_i_)*

***Table 9. Parameters and results for model 6***

| MODEL 6 | | | | |
| --- | --- | --- | --- | --- |
| Parameters | | **Value** | | |
| Dependent variable | | *ARRS_i_* | | |
| Independent variables | | *CC_i_; SVMB_i_; MV_(t-1)_* | | |
| Maximum number of basis functions | | 14 | | |
| Order of interaction | | 2 | | |
| Penalty | | 2 | | |
| Threshold | | 0.0005 | | |
| Removal of irrelevant basis functions | | Yes | | |
| Number of observations | | 219 | | |
| Basis function | | ***β*** | ***t* statistics** | ***p*** |
|  | Constant | 0.240 (0.015) | 14.511*** | 0.0000 |
| BF1 | max(0, *CC_i_* – 0.714) | 0.085 (0.064) | 1.474* | 0.0492 |
| BF2 | max(0, 0.714 – *CC_i_*) | -0.169 (0.042) | -6.105*** | 0.0000 |
| BF3 | max(0, *SVMB_i_* – 0.033) | 0.710 (0.152) | 4.528*** | 0.0000 |
| BF4 | max(0, 0.033 – *SVMB_i_*) | -6.612 (0.384) | -9.887*** | 0.0000 |
| BF5 | max(0, *MV_(t-1_*_)_ – 0.076) | -0.331 (0.083) | -3.732*** | 0.0002 |
| BF6 | max(0, 0.076 – *MV_(t-1)_*) | 2.776 (0.363) | 6.048*** | 0.0000 |
| BF7 | max(0, 0.033 – *SVMB_i_*) ∙ max(0, *MV_(t-1_*_)_ – 0.027) | 19.963 (0.379) | 1.858* | 0.0376 |
| Results | | **Value** | | |
| GCV | | 0.0051 | | |
| *R^2^* | | 0.7273 | | |
| Adjusted *R^2^* | | 0.7170 | | |

standard error in parentheses; ***, ** and * denote significant levels at 0.1%, 1%, and 5%, respectively

*The graphic presentation of MARS model 6 is shown in Fig A4 (S1 File. Supporting information).*

*In summary, the robustness checks carried out demonstrated that the main findings are robust to the revised research sample in the form of a limitation to the 250 largest companies in the S&P500 index*”.

**page 49:**

**Supporting information list:**

**S6 File. Data set**

**is revised into,**

**S1 File. Supporting information for: The effect of self-organizing map architecture based on the value migration network centrality measures on stock return. Evidence from the US market.** The Supporting Information, S1 File, contains additional tables and graphs for the robustness analysis.

**S2 File. Data set**

**Comment 2:** “Also, about a decade ago exactly the same market within the same methodology (of MST) has been studies in 'Physical approach to complex systems' doi:10.1016/j.physrep.2012.01.007 (Figs 80-81), and the central hub was the General Electric. It would be interesting to compare and explicitly address this point here.”

**Reply:** The hub nodes comparison has been added on **page 23**.

**Section 3., page 23:**

“*Several previous studies based on data from the US stock market have shown that one company – General Electric (GE) – is the hub vertex and occupies the central position in the correlation-based MST [12,15,66,74,75,107,108] and PMFG [107]. The above results have been confirmed in another study [109], in which two MST-based networks were constructed for the time series of stock returns in 1998-1999 for the 1000 and 100 largest US companies, respectively.*

*In this paper, the American Water Works Company (AWK) is the center of the value migration network, which has the second largest number of sources of value inflow, after Newmont Corporation (NEM);* $k_{i}^{in}$ *is 33 and 36, respectively. General Electric is not found to be the hub, although it is in the inflow phase;* $k_{i}^{in}=2$*. It should be noted that there are significant differences in the construction of the VMN and the cross-correlation network.*”

**Comment 3:** “The corresponding figure 2 of the present manuscript is hardly readable.”

**Reply:** The quality of Figure 2 has been improved.

**Comment 4:** “What makes commenting also difficult is that the equations are not numbered here.”

**Reply:** All the relevant equations have been numbered.

**Response to Reviewer #2:**

I am very grateful to the reviewer for his/her valuable comments, which have guided me in revising the paper. Text changes are marked in yellow. The newly added and significantly revised portions in the paper are also marked in yellow to make them easy to identify. Minor inaccuracies have been found during editing and correction. They have been rectified and marked in blue. All page references are related to the manuscript with the marked changes.

**Comment 1:** “*My main issue is that when building VMN information about market returns seems to be used. I am talking about step 4 (lines 197-198) of the VMN formation algorithm. Doesn't this make results trivial? Annual returns (via market capitalization) inform VMN and then VMN informs annual returns?*”

**Reply:** The yearly logarithmic return of the firm’s market capitalization that indicates the relative change in the company’s market value is used to indicate the direction of the connection between stocks (points 7 and 12 of the VMN building procedure) by assigning the company to the inflow or outflow phase (point 4). However, the assignment to a specific value migration phase occurs indirectly by relating the relative change of company value to the return of market capitalization of the entire market, using the threshold value (points 5 and 6).

The condition for creating a connection between stocks *i* and *j* is as follows:

1) The occurrence of a negative Pearson correlation coefficient between *i* and *j*.
2) Both companies must be assigned to opposite value migration phases, i.e., one in the inflow phase, the other in the outflow phase. In other words, it is impossible to form a connection between *i* and *j* if *i* and *j* are both in the inflow or the outflow phase.

Thus, the construction of the VMN is more complex and depends on more factors than just the market capitalization rate. In addition, four different centrality measures are included in the Kohonen network as the input layer. The output layer of SOM is one of the independent variables in the MARS regression that showed the non-linear effect of the company’s position in the VMN on the annualized rate of return on shares.

**Section 1., page 4:**

“*Furthermore, the degree distribution in the stock network based on mutual information [57] and Engle-Granger cointegration test [58] are also fitted to a power-law model.*

*It has been demonstrated that in- and out-degree distribution in the value migration network (VMN) obeys a power-law [59].*”

**is revised into,**

“*Furthermore, the degree distribution in the stock network based on mutual information [57] and Engle-Granger cointegration test [58] are also fitted to a power-law model.*

*The value migration network (VMN) is a network built on the basis of negative correlations between returns of daily stock price and the relative changes in the firms’ market capitalization. In the VMN, companies (stocks) are represented as nodes, and the value flows between stocks are modeled as edges. It has been demonstrated that in- and out-degree distribution in the VMN obeys a power-law* (Siudak 2022)*.*”

**Section 2., page 8:**

“*First, I constructed the VMN (the appropriate network construction procedure is presented in section 2.1).*”

**is revised into,**

“*First, I constructed the VMN based on i) negative Pearson correlation coefficients, used for identification connections between assets, and ii) the relative changes in market capitalization of each stock utilized to form the final direction of the relationship in the directed graph. The appropriate network construction procedure is presented in Section 2.1.*

**Section 2.1., pages 12-13, the last sentence of Section 2.1:**

The following has been added: “*It should be emphasized that creating a connection between a pair of stocks in the VMN is possible under two conditions: i) the occurrence of a negative Pearson correlation coefficient among pair of stocks; ii) both assets have to be assigned to opposite phases of value migration. It is not possible to form a connection between two companies if both firms are in the same value migration phase or if there is a positive correlation coefficient between them.*”

**Comment 2:** “*The introduction of the manuscript starts with discussion on VM process (i.e., something dynamic). Yet later in the paper VMN is treated as static. Can't the stocks position in the VMN change over time?*”

**Reply:** The subject of the study is the value migration network as a static network. However, its structure takes into account the value migration over one year (between the end of 2018 and the end of 2019). Nevertheless, the Reviewer’s comment is relevant; hence this issue was emphasized as a proposal for further research.

**Section 5., page 40, the last sentence of manuscript:**

The following has been added: “*Further research is needed to investigate value migration in a dynamic approach, where the subject of the study is a dynamic network. Such a network pattern will enable the analysis of the intensity of changes in the value migration process. However, parameters such as the window length and the rolling step must be properly set to use the rolling window approach for the value migration network.*”

**Comment 3:** “*Initial paragraphs of Section 2 are written in confusing manner. It is not fully clear what is used as input for VMN construction and what is the output. Similarly it is not clear what is the input for Kohonen network and how should we interpret its output. More emphasis on "input"->"process"->"output" should be made, so that it would be clear for readers unfamiliar with specificities of each process.*”

**Reply:** Thank you for that suggestion. The initial paragraphs of Section 2 have been improved.

**Section 2., page 8:**

“*First, I constructed the VMN (the appropriate network construction procedure is presented in section 2.1). Then I employed centrality measures (in- and out-degree; entropy; HITS centrality – see details in section 2.5) to detect the VMN topology based on Kohonen network (section 2.2). This topology was used to classify companies into disjoint groups taking into con-sideration specified neurons, using the entire data set (training, test, and validation set). Then I sorted the groups (neurons) in a non-decreasing order according to the average annualized log-rate of return on shares (ARRS) and assigned ranks also in a non-decreasing manner. Finally, MARS regression was estimated to analyze relationships between the firm’s structural position in the value migration network and the rate of return on shares (see de-tails in Section 2.3). The dependent variable and independent variables are discussed in Sections 2.4 and 2.5, respectively*.”

**is revised into,**

“*First, I constructed the VMN based on i) negative Pearson correlation coefficients, used for identification connections between assets, and ii) the relative changes in market capitalization of each stock utilized to form the final direction of the relationship in the directed graph. The appropriate network construction procedure is presented in Section 2.1. The created value migration network is the basis for generating variables to search for the consequences of the network position of the enterprise and the network structure. Centrality measures (in- and out-degree; entropy; HITS centrality – see details in Section 2.5), taking into account the multifaceted position of the company in the network, were adopted as network variables.*

*Then I employed these centrality measures as the input variables in the input layer of SOM to detect the VMN topology based on the Kohonen network (Section 2.2). This topology was used to classify companies into disjoint groups taking into consideration specified neurons, using the entire data set (training, test, and validation set). The output layer of the Kohonen network consists of clusters with assigned companies due to the similarity according to the centrality measures. Then I sorted the groups (neurons) in a non-decreasing order according to the average annualized log-rate of return on shares (ARRS) and assigned ranks also in a non-decreasing manner. As a result, an independent variable (clusters of centrality) is created. Finally, MARS regression was estimated to analyze relationships between the firm’s structural position in the value migration network and the rate of return on shares (see details in Section 2.3). The dependent variable and independent variables used in the MARS regression are discussed in Sections 2.4 and 2.5, respectively.*”

**Comment 4:** “*Is daily return data sufficient for this task? What would change if the data would be of higher/lower frequency?*”

**Reply:** The adopted *daily return frequency* results from the need to capture the actual value migration. Daily returns are the most common approach adopted in correlation-based network analysis. A similar approach has been proposed for constructing the value migration network. The relationships between stocks and their directions are determined based on daily rates of return and relative annual changes in the market value. The period adopted for the rate of return should not be too long (lower frequency). To the best of my knowledge, the lower frequency than daily return is not found in the literature. For example, the weekly stock returns would only be 52 data per year (that is not enough). This approach would also mean that important events in the financial market could be omitted. However, the frequency should not be too high in order to eliminate short-term fluctuations in stock prices. For example, for a 5 or 10 minutes frequency, the speculative motive might be biased. This effect should be minimized when building the VMN.

**Comment 5:** “*Most figures are unreadable. Resolution is extremely low - can't make out what is on the axes.*”

**Reply:** The quality of all Figures has been improved.

**Comment 6:** “*It seems that term "multidimensional centrality" is used somewhat confusingly. Do you mean "network centrality" in the VMN? Which of the centrality measures do you have in mind, or the results do not depend on the centrality measure? Typically "multidimensional centrality" is centrality of a node in multidimensional (multilayer) network, but here we have no such networks.*”

**Reply:** Thank you for pointing this out. In fact, the term multidimensional centrality refers to vertex centrality in multilayer networks. The relevant parts of the text have been corrected.

**Section 1., page 6:**

**“***(…) instead, a self-organizing map (SOM) of value migration based on the multidimensional central position of the enterprise in the VMN has been created.***”**

**is revised into,**

**“***(…) instead, a self-organizing map (SOM) of value migration based on the central position of the enterprise in the VMN has been created.***”**

**Section 1., page 7:**

**“***Secondly, I employ MARS regression models to examine the impact of a self-organizing map in terms of the multidimensional centrality position in the VMN on the annualized stock’s returns.***”**

**is revised into,**

**“***Secondly, I employ MARS regression models to examine the impact of a self-organizing map in terms of the centrality position in the VMN on the annualized stock’s returns.***”**

**Section 4., page 23:**

**“***This section reports the impact of multidimensional centrality in VMN on the annualized stock’s return.***”**

**is revised into,**

**“***This section reports the impact of centrality measures of the VMN on the annualized stock return.***”**

**Section 5., page 38:**

**“***Specifically, the investigation was focused on whether the multidimensional centrality of the company in the VMN affects the annualized rate of return on shares.***”**

**is revised into,**

**“***Specifically, the investigation was focused on whether the central position of the company in the VMN affects the annualized rate of return on shares.***”**

**Section 5., page 38:**

**“***This study appears to be the first to demonstrate that value migration, measured by means of a self-organizing map based on the multidimensional centrality of the company in the VMN, plays an important role in determining stock’s return levels.***”**

**is revised into,**

**“***This study appears to be the first to demonstrate that value migration, measured by means of a self-organizing map based on the centrality measures of the VMN, plays an important role in determining stock’s return levels.***”**

**Comment 7:** “*Lines 69-70: VMN hasn't yet been introduced, but we are already discussing its properties. At this point it is hard to comprehend what does it imply for the VMN network to have power-law degree distribution.*”

**Reply:** Thank you kindly for your suggestion. A brief introduction to the value migration network has been added.

**Section 1., page 4:**

“*Furthermore, the degree distribution in the stock network based on mutual information [57] and Engle-Granger cointegration test [58] are also fitted to a power-law model.*

*It has been demonstrated that in- and out-degree distribution in the value migration network (VMN) obeys a power-law [59].*”

**is revised into,**

“*Furthermore, the degree distribution in the stock network based on mutual information [57] and Engle-Granger cointegration test [58] are also fitted to a power-law model.*

*The VMN is a network built on the basis of negative correlations between returns of daily stock price and the relative changes in the firms’ market capitalization. In the VMN, companies (stocks) are represented as nodes, and the value flows between stocks are modeled as edges. It has been demonstrated that in- and out-degree distribution in the value migration network (VMN) obeys a power-law* (Siudak 2022)*.*”

**Section 1., page 6:**

“*(…) methods in one study: 1) complex network, 2) artificial neural networks (ANN), and 3) multivariate adaptive regression splines (MARS). The VMN is a network built on the basis of negative correlations between returns of daily stock price and changes in the firms’ market capitalization. This work shows that the topological position in the value migration network (…).*”

**is revised into,**

*“(…) methods in one study: 1) complex network, 2) artificial neural networks (ANN), and 3) multivariate adaptive regression splines (MARS). This work shows that the topological position in the value migration network (…).*”

**Comment 8:** “*Lines 77-78: Requires citation?*”

**Reply:** The required citations, described and grouped in more detail, are provided in the same and the next paragraph – pages 3-4. order to avoid an unnecessary double citation, a minor correction has been made.

**Section 1., page 4:**

“*Many scientists have recently discussed the question of which stocks are superior – central or peripheral?*”

**is revised into,**

“*Some studies in this area have recently discussed which stocks are superior – central or peripheral.”*

**Comment 9:** “*Lines 157-159: Sentence makes no sense. How can measures perform? What do they perform?”*

**Reply:** This sentence has been reformulated.

**Section 2., page 8:**

**“***Then I employed centrality measures (in- and out-degree; entropy; HITS centrality – see details in section 2.5) to perform the VMN topology based on Kohonen network (section 2.2)*”.

**is revised into,**

“*Then I employed these centrality measures as the input variables in the input layer of SOM to detect the VMN topology based on the Kohonen network (Section 2.2)*.”

**Comment 10:** “*Lines 178-188: MTS -> MST. This change likely will need to be made in other parts of the manuscript, too”*.

**Reply:** Thank you very much for noticing a slight typing error. All these errors have been corrected (see pages 9 and 38 of the manuscript with marked changes).

**Comment 11:** “*Line 202: Why? Wouldn't it be more reasonable to use unit threshold value?*”

**Reply:** Generally, one could assume that the company is assigned to the inflow phase if $R_{{MV}_{i}}\left( t \right)\geq0$ and to the outflow phase if$R_{{MV}_{i}}\left( t \right)<0$. However, such a division would mean that companies with a very small increase in market value $\left( R_{{MV}_{i}}\left( t \right)\sim0 \right)$ would be assigned to the inflow phase. Hence, the concept of applying the threshold value $\left( \theta{\cdot R}_{\sum{MV}_{i}\left( t \right)} \right)$. The use of a unit threshold value $\left( \theta=1 \right)$ would be too restrictive. Companies with a significant increase in the change in market value but slightly smaller than the change in capitalization for the entire market would be assigned to the outflow phase.

It seems that assuming the threshold value at the level of 0.6667 is a suitable compromise between the above possibilities, i.e., the inflow value includes companies with a positive relative change in market value, but this change must be significant enough to reach the level of at least 2/3 (66.67%) of the change in capitalization for the entire market $\left( >0,6667{\cdot R}_{\sum{MV}_{i}\left( t \right)} \right)$.

**Comment 12:** “*Line 207: Is it possible that both i and j are in inflow phase? How can this happen? What does it mean?*”

**Reply:** It is impossible that both *i* and *j* are in the inflow (or outflow) phase at the same time. In step 6 of the VMN build procedure, each stock is assigned to one of the two possible phases, inflow or outflow, where the stock is assigned to the inflow phase: if $R_{{MV}_{i}}\left( t \right)\geq\theta R_{\sum{MV}_{i}\left( t \right)}$ or to the outflow phase: if$R_{{MV}_{i}}\left( t \right)<\theta R_{\sum{MV}_{i}\left( t \right)}$. On this basis, the variable x_ij_ will take the value 1 if (see step 7):

$R_{{MV}_{i}}\left( t \right)\geq\theta R_{\sum{MV}_{i}\left( t \right)} and R_{{MV}_{j}}\left( t \right)<\theta R_{\sum{MV}_{j}\left( t \right)}$ (the flow of value from *j* to *i*)

or the value -1 if $R_{{MV}_{i}}\left( t \right)<\theta R_{\sum{MV}_{i}\left( t \right)} and R_{{MV}_{j}}\left( t \right)\geq\theta R_{\sum{MV}_{j}\left( t \right)}$ (the flow of value from *i* to j)

Any other possibility, i.e.

$R_{{MV}_{i}}\left( t \right)\geq\theta R_{\sum{MV}_{i}\left( t \right)} and R_{{MV}_{j}}\left( t \right)\geq\theta R_{\sum{MV}_{j}\left( t \right)}$

or

$R_{{MV}_{i}}\left( t \right)<\theta R_{\sum{MV}_{i}\left( t \right)} and R_{{MV}_{j}}\left( t \right)<\theta R_{\sum{MV}_{j}\left( t \right)}$

means that both *i* and *j* are in the same phase: inflow or outflow, and the variable x_ij_ takes the value 0, so the relation *i* -> *j* or *j* --> *i* is not considered further (see the final step (12)).

**Section 2.1., page 11:**

“*Each stock is assigned to one of the two possible phases, (1) inflow and (2) outflow, by means of the following formulas: inflow phase: if* $R_{{MV}_{i}}\left( t \right)\geq\theta R_{\sum{MV}_{i}\left( t \right)}$ *and outflow phase: if*$R_{{MV}_{i}}\left( t \right)<\theta R_{\sum{MV}_{i}\left( t \right)}"$.

**is revised into,**

“*Each stock is assigned to one of the two possible phases, (1) inflow or (2) outflow, by means of the following formulas: inflow phase: if* $R_{{MV}_{i}}\left( t \right)\geq\theta R_{\sum{MV}_{i}\left( t \right)}$ *and outflow phase: if*$R_{{MV}_{i}}\left( t \right)<\theta R_{\sum{MV}_{i}\left( t \right)}"$.

**Comment 13:** “Line 436: Figure seems to contain undirected network, yet the text seems to imply that VMN is directed network. Or is it poor quality of the figure?”

**Reply:** The value migration network is a directed network. The quality of Figure 2 has been improved.

**Section 3 page 22, caption of Fig 2.**

“*Fig 2. Value migration network (visualization algorithm: Fruchterman & Reingold [104]; performed in NetMiner [105])*”

**is revised into,**

“Fig 2. Value migration network. Green nodes denote companies in the inflow stage; red vertices indicate firms in the outflow stage; black nodes are isolated (visualization algorithm: Fruchterman & Reingold [104]; performed in NetMiner [105])

**Comment 14:** “*What do you mean by "error level". Your data seems to be unlabeled and you are using unsupervised machine learning algorithm. How do you define error?*”

**Reply:** The term “error level” has indeed been inadvertently used. The quantization error is used to evaluate the quality of unsupervised learning network algorithms. This error measures the average distance between the input values of the input layer and the weights of the corresponding winning neuron. The quantization error evaluates the fit of the topology map to the data. The smaller the error, the better the fit of the topological layer to the data.

**Section 2.2., page 15:**

“*The research sample was divided into three sets of data in a proportion of 70% for the training set, 15% for the test set, and 15% for the validation set. The training set is employed to construct the Kohonen network, the test set – to evaluate the quality of the classification as the ability to generalize, while the validation set – to verify the applicability of the created SOM model in the network training process.*”

**is revised into,**

“*The research sample was divided into three sets of data in a proportion of 70% for the training set, 15% for the test set, and 15% for the validation set. The training set is employed to construct the Kohonen network, the test set – to evaluate the quality of the classification as the ability to generalize, while the validation set – to verify the applicability of the created SOM model in the network training process. The SOM quality evaluation is performed using the quantization error, which measures the mean distance between each data case and its corresponding winning neuron*

$Q_{error}=\frac{1}{N}\sum_{i=1}^{n} \left( x_{i}^{k}-w_{c,i} \right)^{2}$	*(23)*

*where:* $w_{c,i}$ *– weight of the winning neuron corresponding to the input value of the ith input node.*

*The quantization error assesses the fit of the topology map to the data. The best result of the Kohonen network algorithm is yielded for the minimum quantization error value.*”

**Section 4., page 24, Table 1:**

| Network Id | Network* | Topological layer size | Error level | | |
| --- | --- | --- | --- | --- | --- |
|  |  |  | **training** | **test** | **validation** |
| 1 | SOM 4-9 | 3x3 | 0.00444 | 0.00318 | 0.00301 |
| 2 | **SOM 4-12** | **3x4** | **0.00308** | **0.00319** | **0.00225** |
| 3 | SOM 4-16 | 4x4 | 0.00173 | 0.00347 | 0.00311 |
| 4 | SOM 4-20 | 4x5 | 0.00132 | 0.00377 | 0.00359 |
| 5 | SOM 4-25 | 5x5 | 0.00078 | 0.00491 | 0.00385 |
| 6 | SOM 4-15 | 3x5 | 0.00217 | 0.00324 | 0.00297 |
| 7 | SOM 4-18 | 3x6 | 0.00141 | 0.00367 | 0.00335 |
| 8 | SOM 4-24 | 4x6 | 0.00113 | 0.00405 | 0.00384 |
| 9 | SOM 4-30 | 5x6 | 0.00044 | 0.00439 | 0.00309 |
| 10 | SOM 4-36 | 6x6 | 0.00060 | 0.00442 | 0.00333 |

**is revisited into,**

| Network Id | Network* | Topological layer size | Quantization error | | |
| --- | --- | --- | --- | --- | --- |
|  |  |  | **training** | **test** | **validation** |
| 1 | SOM 4-9 | 3x3 | 0.00444 | 0.00318 | 0.00301 |
| 2 | **SOM 4-12** | **3x4** | **0.00308** | **0.00319** | **0.00225** |
| 3 | SOM 4-16 | 4x4 | 0.00173 | 0.00347 | 0.00311 |
| 4 | SOM 4-20 | 4x5 | 0.00132 | 0.00377 | 0.00359 |
| 5 | SOM 4-25 | 5x5 | 0.00078 | 0.00491 | 0.00385 |
| 6 | SOM 4-15 | 3x5 | 0.00217 | 0.00324 | 0.00297 |
| 7 | SOM 4-18 | 3x6 | 0.00141 | 0.00367 | 0.00335 |
| 8 | SOM 4-24 | 4x6 | 0.00113 | 0.00405 | 0.00384 |
| 9 | SOM 4-30 | 5x6 | 0.00044 | 0.00439 | 0.00309 |
| 10 | SOM 4-36 | 6x6 | 0.00060 | 0.00442 | 0.00333 |

**Comment 15:** “*Line 681-684: What does it mean for a firm to have a positive impact on stock's return? Confusing sentence.*”

**Reply:** This means that regardless of the initial value of *CC_i_*, companies in the cluster with a higher index (*CC_i_*=6 or 7 or 8) have a higher rate of return on shares. In other words, if *CC_i_* is greater than 5, then the greater the value of *CC_i_*, the greater the *ARRS_i_* and vice versa (a positive effect in terms of regression results). For companies from clusters 1-4 (*CC_i_*=1, 2, 3, or 4), the greater the *CC_i_* value, the smaller the decrease in *ARRS_i_*, and if the smaller the *CC_i_* value, the greater the decrease in *ARRS_i_* (negative effect). The statement indicated by the Reviewer was reformulated.

**Section 5., pages 38-39:**

“*Firms classified into neurons (3,2); (3,1) and (2,1) – with CC_i_ at 6, 7, or 8 – have a positive impact on stock’s return and companies from neurons (2,4); (1;4); (3,4); (1,3) – with CC_i_ at 1, 2, 3, or 4 – have a negative effect”*.

**is revised into,**

“*For firms classified into neurons (3,2); (3,1), and (2,1) – with CCi at 6, 7, or 8 – the company’s place on the self-organizing feature map in terms of its central position in the VMN has a positive effect on stock’s return and for companies from neurons (2,4); (1;4); (3,4); (1,3) – with CCi at 1, 2, 3, or 4 – a negative effect is demonstrated”*.
