# Supplementary material for: The effect of self-organizing map architecture based on the value migration network centrality measures on stock return. Evidence from the US market
Source: PLoS One. 2022 Nov 1;17(11):e0276567. doi: 10.1371/journal.pone.0276567 (PMC9624434; doi:10.1371/journal.pone.0276567)
Supplement: Attachment — Submitted filename: Detailed Response to Reviewer_2.docx [file pone.0276567.s004.docx]

**Detailed Response to Reviewers**

**Response to Academic Editor**

October 04, 2022

*Dear Academic Editor,*

Thank you very much for the referee report on my manuscript PONE-D-22-15643 entitled “The effect of self-organizing map architecture based on the value migration network centrality measures on stock return. Evidence from the US market”.

Please find enclosed a detailed, point-to-point response to all Reviewer comments with all changes clearly specified. Changes in the text are marked with colors, as follows:

Reviewer #1: first round of review: green; second round: grey;

Reviewer #2: first round of review: yellow.

Minor inaccuracies have been found during editing and correction. They have been rectified and marked in blue. All page references are related to the manuscript with the marked changes.

I wish to take this opportunity to thank the Reviewers for their time, effort, and insightful comments that have resulted in an improved manuscript. I would also like to thank you for handling my paper. Hopefully, my revised manuscript can be regarded as meeting the high standards of PLOS ONE journal.

With kind regards

**Point-to-point responses to the Reviewers**

**Response to Reviewer #1:**

I am grateful for your suggestions to improve my manuscript. In my new version, I have followed your recommendations. Changes in the text are marked with colors, as follows:

first round of review: green;

second round of review: grey.

All page references are related to the manuscript with the marked changes. I hope my revised manuscript meets your high standards.

**Comment:** “*The Author's response concerning my quantitative arguments related to the degeneracy of so-constructed correlation matrix expressed in my previous report is not satisfactorily expressed. This issue is now addressed, but as far as I can see only in the response letter and not in the manuscript, and, in addition, it is too descriptive. This issue should be made more quantitative and addressed in the manuscript text. In particular, does the claim that reducing the number of S&P500 companies used to the 250 largest ones does not change the result shown does mean that the remaing ones are disonnected from those 250 largest ones? If so, what for to include them?*”

**Reply:** One of the limitations of the study is the imperfect projection of the full complexity, which was highlighted in the previous review. The goal of the additional analysis was to assess whether the indicated limitation causes a distortion of the basic results. These analyzes are presented in Section 5 and in the Appendix (S1 File. Supporting information).

The reduction of the analyzed companies to TOP250 in terms of market capitalization did not change the results in relation to all 498 companies. This does not mean, however, that value migration takes place severally and independently of each other in the group of only the largest and smallest stocks. Value migration can be analyzed in relation to a specified system of companies, limited do the entire market or its selected part (e.g., TOP100 or TOP250). However, the inclusion of all 498 companies was intended to capture the wide-ranging process of value migration in the financial market.

Section 5 has been extended at the beginning and end of this section.

**Pages 35-38:**

***“5. Additional analysis***

*In this section, the robustness of obtained results is carried out to narrow the research sample to the 250 largest companies from the S&P500 index. The largest companies in terms of market capitalization in 2019. The analysis period remains the same; therefore, the dataset consists of 253 daily closing prices in every 250 time series. The value migration network for the 250 largest companies is presented in Figure A1, available in Supporting Information, S1 File. The largest component of the VMN contains 219 firms.*

*(…)*

*In summary, the robustness checks carried out demonstrated that the main findings are robust to the revised research sample in the form of a limitation to the 250 largest companies in the S&P500 index*.”

**is revised into,**

**“5. Additional analysis**

The limitation of the VMN built for 498 stocks is the imperfect projection of the full complexity for such a number of companies in the adopted one-year period. The value migration process should be analyzed over a properly defined period. It should not be too short in order to eliminate short-term fluctuations in stock prices. However, it should not be too long either to capture the scale of the process of changing the value of companies. The adopted period of one year results from the need to capture the current value migration process. In this section, the robustness of obtained results is carried out to narrow the research sample to the 250 largest companies from the S&P500 index. A similar approach was used in the study [110] to investigate the statistical properties of price returns for the 100 largest companies on the NYSE in one year, 2002. The aim of the analysis is to assess whether the above limitation biases the obtained results.

The largest companies are considered in terms of market capitalization in 2019. The analysis period remains the same; therefore, the dataset consists of 253 daily closing prices in every 250 time series. The value migration network for the 250 largest companies is presented in Figure A1, available in Supporting Information, S1 File. The largest component of the VMN contains 219 firms.

The obtained topological layer of the SOM with the assumed dimensions of 3 x 4 again contains eight disjoint groups. The number of companies assigned to individual clusters is presented in Table A1 and Fig A2a (see S1 File. Supporting information). The calculation of the cluster of centrality (CC_i_) variable is provided in Table A2 (see S1 File. Supporting information).

The results of the MARS regression without the interaction of variables (model 5) are presented in Table 8. The final regression equation of model 5 is as follows:

ARRS_i_ = 0.221 + 0.085 ∙ max(0, CC_i_ – 0.714) – 0.264 ∙ max(0, 0.714 – CC_i_) + 1.747 ∙

(43)

max(0, SVMB_i_ – 0.033) – 3.504 ∙ max(0, 0.033 – SVMB_i_) – 0.227 ∙ max(0, MV_(t-1)_ – 0.076) + 1.925 ∙ max(0, 0.076 – MV_(t-1)_) – 1.267 ∙ max(0, SVMB_i_ – 0.064)

The graphical presentation of model 5 is demonstrated in Fig A3 (S1 File. Supporting information).

**Table 8. Parameters and results for model 5**

| MODEL 5 | | | | |
| --- | --- | --- | --- | --- |
| Parameters | | **Value** | | |
| Dependent variable | | ARRS_i_ | | |
| Independent variables | | CC_i_; SVMB_i_; MV_(t-1)_ | | |
| Maximum number of basis functions | | 14 | | |
| Order of interaction | | 1 | | |
| Penalty | | 2 | | |
| Threshold | | 0.0005 | | |
| Removal of irrelevant basis functions | | Yes | | |
| Number of observations | | 219 | | |
| Basis function | | **β** | **t statistics** | **p** |
|  | Constant | 0.221 (0.013) | 16.854*** | 0.0000 |
| BF1 | max(0, CC_i_ – 0.714) | 0.085 (0.063) | 1.343* | 0.0487 |
| BF2 | max(0, 0.714 – CC_i_) | -0.264 (0.042) | -5.834*** | 0.0000 |
| BF3 | max(0, SVMB_i_ – 0.033) | 1.747 (0.666) | 2.622** | 0.0094 |
| BF4 | max(0, 0.033 – SVMB_i_) | -3.504 (0.408) | -8.592*** | 0.0000 |
| BF5 | max(0, MV_(t-1)_ – 0.076) | -0.227 (0.061) | -3.696*** | 0.0003 |
| BF6 | max(0, 0.076 – MV_(t-1)_) | 1.925 (0.298) | 6.466*** | 0.0000 |
| BF7 | max(0, SVMB_i_ – 0.064) | -1.267 (0.675) | -1.877* | 0.0491 |
| Results | | **Value** | | |
| GCV | | 0.0061 | | |
| R^2^ | | 0.6755 | | |
| Adjusted R^2^ | | 0.6631 | | |

standard error in parentheses; ***, ** and * denote significant levels at 0.1%, 1%, and 5%, respectively

Model 5 is the counterpart of Model 1. By comparing both models, it can be seen that the structure of all the spline basis functions and the signs (positive/negative) of the regression coefficients are preserved. Furthermore, all regression coefficients of the contained spline basis functions are statistically significant (p<0.05). The value of the knot point of BF1 and BF2 is the same as in the original model: the value of 0.714 normalized knot; the value of 5 CC_i_ variable. This means that the critical threshold of the cluster for the non-linear effect remains unchanged. However, due to the change in the research sample, the knot point values have changed for the remaining functions related to the SVMB_i_ and MV_(t-1)_ variables.

The results of Model 6 with second-order interactions between predictors are shown in Table 9.

**Table 9. Parameters and results for model 6**

| MODEL 6 | | | | |
| --- | --- | --- | --- | --- |
| Parameters | | **Value** | | |
| Dependent variable | | ARRS_i_ | | |
| Independent variables | | CC_i_; SVMB_i_; MV_(t-1)_ | | |
| Maximum number of basis functions | | 14 | | |
| Order of interaction | | 2 | | |
| Penalty | | 2 | | |
| Threshold | | 0.0005 | | |
| Removal of irrelevant basis functions | | Yes | | |
| Number of observations | | 219 | | |
| Basis function | | **β** | **t statistics** | **p** |
|  | Constant | 0.240 (0.015) | 14.511*** | 0.0000 |
| BF1 | max(0, CC_i_ – 0.714) | 0.085 (0.064) | 1.474* | 0.0492 |
| BF2 | max(0, 0.714 – CC_i_) | -0.169 (0.042) | -6.105*** | 0.0000 |
| BF3 | max(0, SVMB_i_ – 0.033) | 0.710 (0.152) | 4.528*** | 0.0000 |
| BF4 | max(0, 0.033 – SVMB_i_) | -6.612 (0.384) | -9.887*** | 0.0000 |
| BF5 | max(0, MV_(t-1)_ – 0.076) | -0.331 (0.083) | -3.732*** | 0.0002 |
| BF6 | max(0, 0.076 – MV_(t-1)_) | 2.776 (0.363) | 6.048*** | 0.0000 |
| BF7 | max(0, 0.033 – SVMB_i_) ∙ max(0, MV_(t-1)_ – 0.027) | 19.963 (0.379) | 1.858* | 0.0376 |
| Results | | **Value** | | |
| GCV | | 0.0051 | | |
| R^2^ | | 0.7273 | | |
| Adjusted R^2^ | | 0.7170 | | |

standard error in parentheses; ***, ** and * denote significant levels at 0.1%, 1%, and 5%, respectively

Model 6 is the counterpart of Model 2. It consists of fewer base functions, six functions related to three variables, and one function with a second-order interaction. The following basic functions of model 6 (BF1÷BF7) are equivalent to the basic functions of BF1, BF2, BF3, BF4, BF9, BF10, and BF8 of model 2, respectively. The construction of all basis functions and the signs of the regression coefficients are preserved and statistically significant at the significance level of 5%. In this case, the value of the knot point of BF1 and BF2 is the same as in the original model 2 and for models 1 and 5.

The final regression equation of model 5 is as follows:

ARRS_i_ = 0.240 + 0.085 ∙ max(0, CC_i_ – 0.714) – 0.169 ∙ max(0, 0.714 – CC_i_) + 0.710 ∙ max(0, SVMB_i_ – 0.033) – 6.612 ∙ max(0, 0.033 – SVMB_i_) – 0.331 ∙ max(0, MV_(t-1)_ – 0.076) + 2.776 ∙ max(0, 0.076 – MV_(t-1)_) + 19.963 ∙ max(0, MV_(t-1)_ – 0.027) ∙

(44)

max(0, 0.033 – SVMB_i_)

The graphic presentation of MARS model 6 is shown in Fig A4 (S1 File. Supporting information).

In summary, the robustness checks carried out demonstrated that the main findings are robust to the revised research sample in the form of a limitation to the 250 largest companies in the S&P500 index. Value migration can be analyzed in relation to a specified system of enterprises, e.g., the entire market, its selected part, or industry. The results obtained for the group of 250 largest companies are consistent with those for all companies from the S&P500 index. However, this does not mean that the value flow occurs separately within one of the two groups, the largest or the smallest companies. In other words, it is possible that the value flows between a company assigned to the TOP 250 group and smaller ones. However, the VMN built on the basis of all 498 stocks covers a wider range of value migration in the financial market.”

**page 49-50:**

One reference has been added:

110.	Coronnello C, Tumminello M, Lillo F, Miccichè S, Mantegna RN. Economic sector identification in a set of stocks traded at the New York Stock Exchange: a comparative analysis. Noise and Stochastics in Complex Systems and Finance. 2007. p. 66010T. doi:10.1117/12.729619

**page 50:**

**Supporting information list:**

**S6 File. Data set**

**is revised into,**

**S1 File. Supporting information for: The effect of self-organizing map architecture based on the value migration network centrality measures on stock return. Evidence from the US market.** The Supporting Information, S1 File, contains additional tables and graphs for the robustness analysis.

**S2 File. Data set**
